# Negative impact of family religious and spiritual beliefs in schizophrenia – a 2 year follow-up case

**DOI:** 10.1192/j.eurpsy.2021.1810

**Published:** 2021-08-13

**Authors:** J. Mauricio, M. Albuquerque

**Affiliations:** 1 Mental Health, ULSAM, VIANA DO CASTELO, Portugal; 2 Mental Health, Hospital de Cascais, Alcabideche, Portugal

**Keywords:** religious beliefs, treatment adherence, schizophrénia, cultural

## Abstract

**Introduction:**

For individuals with mental disorders and their families, religion and spirituality may have a significant influence over how these conditions are understood, managed and treated. Family can act as a moderator in which psychotic patients interpret and explain internalized events. However, they can have a negative impact when discouraging diagnosis and treatment adherence.

**Objectives:**

Explore the impact of family religious and spiritual beliefs on clinical outcomes among a schizophrenic patient. Investigate the psychiatrist’s role in addressing barriers to treatment adherence.

**Methods:**

Data retrieved from clinical interview. Subsequent non-systematic review of the most relevant literature on the topic.

**Results:**

We report a case of a 30-year-old single catholic woman, living with her parents. She had a past history of psychotic symptoms that were interpreted in a context of a depressive episode. After some months she fulfilled the criteria for Schizophrenia and anti-psychotic was prescribed. Family always demonstrated doubts about the disease and negatively influenced the treatment adherence. They believed she was possessed by demons and she was submitted to exorcisms and spiritual therapies. After a 2-year follow-up with erratic treatment regimens and worsening symptoms they accepted her hospitalisation. The majority of symptoms were controlled allowing complete adherence to the same treatment proposed before.

**Conclusions:**

The disease acceptance is a complex process, influenced by multiple beliefs that play different roles in each patient and family, that can adversely influence clinical management. It is essential to understand the family sociocultural environment, by gauging the most influential elements aiming to enhance their compliance with treatment.

**Disclosure:**

No significant relationships.

